# Diagnostic Syllabus of Roetheln (German Measles), Scarlet Fever and Measles

**Published:** 1876-04

**Authors:** J. H. Etheridge

**Affiliations:** Chicago


### DIAGNOSTIC SYLLABUS OF ROETHELN (GERMAN MEASLES), SCARLET FEVER AND MEASLES.

PREPARED BY DR. J. H. ETHERIDGE.

The accompanying syllabus is submitted for the purpose of pointing out the simplest means of differentiating roetheln from the two diseases with which it is easily confounded, of which two diseases some physicians have even gone so far as to say it is a mixture. Clinical observation shows that roetheln may visit the same person more than once. Measles and scarlet fever are *very rarely* met with in the same individual a second time. As a rule, measles protects against future attacks of measles, and having scarlet fever secures the patient against future attacks of scarlet fever. Roetheln protects the individual against neither scarlet fever nor measles; and as it so nearly resembles the two latter and is so often mistaken for them, it is of the highest importance that the differential diagnosis be readily made. The literature of roetheln is exceedingly limited. All the authorities discoverable have been looked up, and they are quoted in the first column of the syllabus. Scarlet fever and measles are so exhaustively treated of by numerous writers that it is thought sufficient to take the description of mild cases from one or two authors only. Roetheln is such a *mild* disease that it is almost always called very mild scarlatina. It calls for little or no treatment. During epidemics of it prevailing in various parts of the country, charlatans are pleased to call it scarlet fever, and credit for skillful and *uniformly successful* treatment of a very serious disease is sure to follow. The practitioner will perceive that *mild* cases of scarlet fever and of measles are described; this is done in order to present, side by side, the three disorders in their greatest degree of similarity.

The salient points of difference are: 1st, *In the temperature;* 2nd, In the mode of the appearance of the eruption; 3rd, *In the characteristic elevation and heightened color of the centre of the patches;* and 4th, In the manner of desquamation.

### *Roetheln, or German Measles.*

1st Stage — Stage of Invasion.

SYMPTOMS.

Slight languor with headache and sometimes nausea and vomiting. In one case clonic convulsions.—*J. L. Smith.*

"Shiverings, nausea, rarely vomiting, itching, redness and pain of eyes with increased lachrymation, sneezing and discharge from nose, cough, sore throat and hoarseness."—*Aitken.*

(In addition to above)—pain in limbs more or less.—*Liveing.*

(In addition to above.) "Sore throat is a most *constant* symptom—is a characteristic feature of the disease occurring in the slightest and most gentle cases."—*Patterson.*

"There is usually sore throat."—*Dunlop.*

"In all cases there is sore throat."—*Murchison.*

DURATION.

Eruption appears on 1st or 2d day, usually the 2d day.—*Liveing, Murchison.*

Appears on 3d or 4th day. — *Aitken, Roberts.*

Appears on 5th day.—*Fox.*

"Some hours, or a day or even a longer duration."—*J. L. Smith.*

### *Scarlet Fever, or Scarlatina.*

1st Stage — Stage of Invasion.

SYMPTOMS.

Chill, vomiting, epistaxis. The fever, increased heat of skin, headache, prostration and general mlaiase vary much in intensity, not including case sof unusual severity.

DURATION.

"Average duration, 24 hours. Eruption usually appears on 2d day, (*i. e.*, at any hour after the 24th hour of sickness). Exceptionally it appears, on the one hand, a few hours after the attack, and, on the other hand, it it may be delayed 1, 2, 3, or more days after the time when it usually appears."—*Flint.*

### *Measles.*

1st Stage — Stage of Invasion.

SYMPTOMS.

Lassitude, shivering, fever, catarrh. The mucous membrane of the eyes, throat, windpipe and bronchial tubes are much affected. Eyes watery, lids puffy; dry cough; hoarseness and difficulty in breathing; drowsiness; great heat of skin; frequent and hard pulse.

DURATION.

"Eruption appears on 4th day—seldom earlier—often later."—*Tanner.*

### *Roetheln, or German Measles.*

### 2nd Stage — Stage of Eruption.

#### MODE OF APPEARANCE.

The eruption appears *all at once over the whole body*—is *sudden and general*—is less marked on the limbs than trunk.—*Aitken, Roberts, Fox, Copeland.*

May first appear upon the back, upon the chest or neck, upon the cheek or upon the forehead; travels downward.—*J. L. Smith.*

#### CHARACTER OF THE ERUPTION.

At first like measles—minute dots, which rapidly assume the appearance of large, irregular shaped patches, varying from three-cent piece to twenty-five cent piece in size.—*Aitken, Liveing, Murchison, Roberts.*

These patches quickly become "raised above the surrounding skin, especially towards the centre of the patch, and are of a darker red color" at the centres.—*Aitken, Roberts, Fox.*

These patches shade off in color towards the margins till the natural color is reached between the patches.—*Aitken.*

The more severe the case the more is the centre of the patches elevated above the surrounding skin.—*Aitken.*

### *Scarlet Fever, or Scarlatina.*

### 2nd Stage — Stage of Eruption.

#### MODE OF APPEARANCE.

Usually appears on body and limbs before it comes out on the face and neck. In exceptional cases it appears first on latter situations.

#### CHARACTER OF THE ERUPTION.

First appearance is in form of dots or specks. These coalesce, forming irregularly distributed patches, which vary in shape and size, having irregular or serrated margins. "In some cases the whole cutaneous surface is covered with efflorescence, presenting the appearance of a boiled lobster."—*Flint.*

Amount of eruption varies very greatly—ranging all the way from slightest possible amount to a bright scarlet hue of the entire surface of the body, no elevation of the skin answering to "centre of patches" is ever noticed. No elevation farther than that we call "goose skin" is ever seen.

### *Measles.*

### 2nd Stage — Stage of Eruption.

#### MODE OF APPEARANCE.

First appears on forehead and face, and gradually extends downwards over the body and legs and arms.

#### CHARACTER OF THE ERUPTION.

Comes out in small circular dots like fleabites. These dots run together and form blotches of a raspberry color, and the latter are very prone to assume a crescentic or horseshoe shape, being slightly elevated above surrounding skin.

Eruption is sometimes diffused over the whole body in a confluent form, and "is of a dull, deep red color, offering a contrast to the crimson or scarlet redness of scarlet fever."—*Flint.*

After a time the patches *may* (*sic!*) all unite, and then the skin becomes to the naked eye of a uniform red color, closely resembling scarlet fever.—*Murchison.*

In two cases out of five, this uniform red color was noticed.—*Liveing.*

DURATION.

"Eruption disappeared in 3 or 4 days." —*Dunlop.*

In proportion to severity: 4 to 10 days. —*Murchison*, *Aitken.*

From 7 to 10 days.—*Liveing.*

"In no case (of 21 reported cases) lasting more than 8 hours."—*Steiner*, of Prague.

"Usually fades in 4 or 5 days."—*Roberts.*

"Fades out 2d or 3d day."—*Fox.*

"Commonly disappeared on the fourth day."—*J. Lewis Smith.*

ACCOMPANIMENTS.

Sore throat is *always* a prominent and troublesome symptom in this stage. This fact is *particularly* emphasized by *Aitken*, *Liveing*, *Murchison*, *Patterson*, *Copeland.*

"In severe cases the hoarseness is so great as frequently to cause entire loss of voice." * * Swelling of the neck ac-

DURATION.

Eruption reaches its maximum of intensity and diffusion on third day after its first appearance. This stage varies from 4 to 6 days. Eruption is usually gone at the end of (5th) fifth day. In some cases it persists a day or two longer.

ACCOMPANIMENTS.

Very numerous. Many of them present in a certain proportion of cases—then again they are all wanting. They vary widely in their dangerousness—some being surely fatal, others being quite innocuous.

There may be sore throat varying from a little redness to a destructive degree of

DURATION.

Begins to fade about the 4th day, in the same order in which it came out.

ACCOMPANIMENTS.

Irritability of the mucous membranes all over the body seems to be a characteristic of measles. Hence bronchitis is almost the rule in this stage.

Redness of the eyes is a constant symptom, amounting to inflammation in some cases.

*Roetheln, or German Measles.*

companies this hoarseness, and is so great that "there is total inability to swallow even the slightest portion of fluid, which generally regurgitates by the nose."—*Aitken.*

Mild inflammation of mucous membrane of throat, mouth, nose and eyes.—*J. L. Smith.*

"There is a combination of scarlatinous angina (sore throat) and tongue, with morbillous (measles) catarrh."—*Murchison.*

"The temperature being always highest on first day of attack, not exceeding 102°, next day falling to 100°, and getting normal on the 5th day.—*Fox.*

"The temperature nearly always *subfebrile* (99.5° to 100.4°)—sometimes febrile (101.3° to 102.2°)."—*Wunderlich.*

"There is scarcely any constitutional disturbance and the temperature is only slightly if at all raised above the normal standard."—*Dunlop.*

"Slight, if any, rise in temperature."—*Steiner.*

Febrile movement constantly mild in uncomplicated cases, ranging from 98° to 100°.—***Reid, J. L. Smith.***

*Scarlet Fever, or Scarlatina.*

inflammation. Watery blebs or blisters sometimes appear on the skin.

Fever rather increases after eruption.

Temperature may reach 105.6° or even a higher point. It usually remains continuously high during the eruption (*Wunderlich*) and it is thus "well distinguished from those affections with which, on account of other symptoms, it is most easily confounded, and more particularly *measles and roetheln.*"—*Wunderlich.*

Pulse may go to 160 or even 170.

No appetite; sometimes vomiting; constipation in some cases—diarrhœa in others; thirst urgent; delirium, common; integument slightly swollen all over, as is shown when patient tries to close the hand.

*Measles.*

Giving a cathartic in measles is most unfortunate, sometimes, because of this sensitiveness of the mucous membranes.

Temperature for *one* day in this stage in uncomplicated cases will rise to 106°. It then rapidly subsides to the normal standard, reaching it in 48 hours.

| 3rd Stage—Stage of Desquamation. | 3rd Stage—Stage of Desquamation. | 3rd Stage—Stage of Desquamation. |
|---|---|---|
| CHARACTER OF THE SCALES. | CHARACTER OF THE SCALES. | CHARACTER OF THE SCALES. |
| Minute portions of cuticle like scales of *fine bran*.<br>Always begins towards centre of the eruptive patch and gradually extends to the circumference.—*Roberts*, *Aitken*, *Murchison*, *Liveing*, *Patterson*, *Dunlop*.<br>"Scales on *hands and feet* are larger, but never reach the size of those of scarlet fever."—*Patterson*. | Comes off in branny scales and in large patches. "Occasionally epidermis of the hands is detached entire, and may be slipped off like a glove. This is true also of the feet.—*Flint*.<br>Sometimes several successive desquamations occur.<br>Frequently accompanied with itching, which in some cases is excessive. | Always in branny scales, not in patches or flakes. |
| DURATION. | DURATION. | DURATION. |
| Five days to 12 or 15 days. | Desquamation usually completed in from 10 to 12 days; exceptionally it continues for several weeks. | From 4 to 8 days. |

